# Severe restless legs syndrome in a family with Alport syndrome

**DOI:** 10.1186/s12882-021-02455-2

**Published:** 2021-07-05

**Authors:** Davide Sparasci, Andrea Rossinelli, Raffaele Ferri, Pietro Cippà, Andrea Rinaldi, Mauro Manconi

**Affiliations:** 1 Sleep Medicine Unit, Neurocenter of Southern Switzerland, Ospedale Civico, Lugano Switzerland; 2 Sleep Research Centre, Oasi Research Institute - IRCCS, Troina, Italy; 3 Division of Nephrology, Ente Ospedaliero Cantonale, Lugano, Switzerland; 4 Institute of Oncology Research, Faculty of Biomedical Sciences, Università della Svizzera italiana, 6500 Bellinzona, TI Switzerland; 5 Faculty of Biomedical Sciences, Università della Svizzera Italiana, Lugano, Switzerland; 6 Department of Neurology, University Hospital, Inselspital, Bern, Switzerland

**Keywords:** Alport syndrome, Restless legs syndrome, Augmentation, Chronic kidney disease

## Abstract

**Background:**

Restless legs syndrome (RLS) is a common sleep-related movement disorder characterized by an urge to move the legs during inactivity, especially at evening-night. RLS is highly prevalent in patients with kidney failure and have an impact on quality of life, mood, sleep quality and overall on compliance to the dialysis. Alport syndrome (AS) is a rare inherited disease, predominantly X-linked, secondary to mutations in genes encoding α3, α4 or α5 chains of type IV collagen, and characterized by hematuria, chronic kidney disease, neurosensory deafness, and lenticonus.

**Case presentation:**

Here we describe a family with a combination of X-linked AS and severe RLS accompanied by periodic limb movements during sleep (PLMS). In the first patient we identified, RLS was complicated by a paradoxical response to dopamine agonists named “augmentation”, leading to sleep disruption, hallucinations and five peritoneal perforations during the peritoneal dialysis due to the difficulty to rest still. Therapeutic adjustments and renal transplantation improved RLS and PLMS. In two brothers, severe RLS prevented a compliance with hemodialysis. Female family members carrying the mutation were also affected by RLS, while those without the mutations were RLS-free.

**Conclusions:**

RLS has not been reported earlier in association with AS, but the peculiar combinations observed in this family will stimulate further clinical studies and motivate nephrologists to seek for RLS symptoms and sleep disturbances in AS patients.

## Background

Alport syndrome (AS) is a rare genetic disorder, predominantly X-linked, with mutations in the genes encoding α3, α4, or α5 chains of collagen IV (COL4A3, COL4A4, and COL4A5) [1]. Since type IV collagen is expressed in several tissues, AS is a multi-organ disease  characterized by hematuria, progressive chronic kidney disease (CKD), neuro-sensorial deafness, and lenticonus. In the most common X-linked form, females carrying the mutation are usually asymptomatic, while males inherit the disease from mothers and develop symptoms with high penetrance with CKD progressing to kidney failure often before the age of 40 years [2].

Restless legs syndrome (RLS) is a common sleep-related sensory-motor disorder, affecting approximately 5 % of the general population in adults, with a female preponderance [3]. RLS is characterized by an uncomfortable urge to move the legs emerging or worsening at rest, mainly at evening/night, which improves or disappears with movement [4]. In moderate-severe cases, RLS impacts sleep, mood, cognition and, in general, quality of life, being as stressful as chronic diseases like diabetes and rheumatoid arthritis [5]. In around 90 % of patients, RLS is accompanied by periodic limb movements during sleep (PLMS) which contribute to sleep fragmentation and in the long-term may increase the risk of cardiovascular disease. RLS can be classified as primary/idiopathic, or secondary/symptomatic if associated to pathological conditions such as iron deficiency, [6] pregnancy, [7] some neurological disorders, [8] and CKD [9].

The prevalence of RLS in CKD exceeds rates of 20–25 %, with a very high prevalence in patients with kidney failure and on dialysis (28.4 %) and a partial resolution after kidney transplantation (6.7 %). RLS is associated with an adverse prognosis and higher mortality in CKD [10]. Compared to RLS free counterparts, hemodialysis patients with RLS are subjects of increased muscle atrophy [11], depression, anxiety, sexual dysfunction, further reduction in quality of life and sleep quality [12–14]. Because of the legs discomfort during periods of inactivity, patients with RLS are twice as likely to leave dialysis early [15]. Dopamine agonists, such as pramipexole, ropinirole and rotigotine, are first line treatments for RLS, which are initially well tolerated and highly effective, even at low dosages and from the first night of administration.

In this paper, we report, for the first time, the association between RLS and AS in a large family, also focusing on the diagnostic, therapeutic and pathophysiological aspects.

## Case presentation

**Fig. 1:**
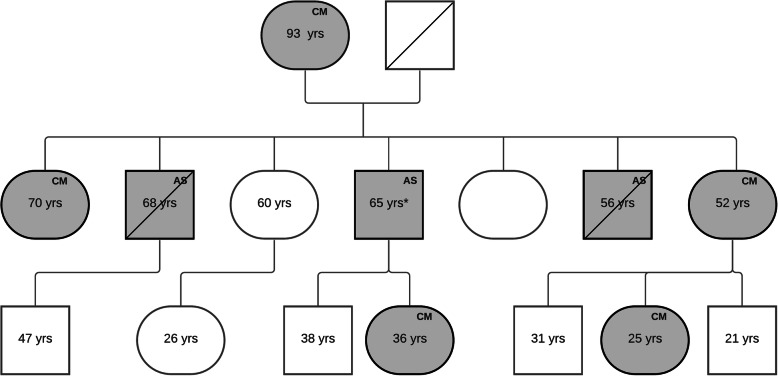
Patient’s family tree. The grey background indicates subjects affected by RLS. AS, male subjects with Alport syndrome. CM, female subjects carrying the mutation. The asterisk identifies the proband. Bars across squares indicate deceased subjects

A 65-year-old man affected by AS presented at the emergency room for psycho-motor agitation and hallucinations, acutely emerged after a severe chronic sleep deprivation, without any previous psychiatric history. He was also affected by RLS since the age of 10 years and, at time of our visit, was treated with pramipexole 0.75 mg/day. He had hearing but not major visual impairment and has been on peritoneal and then on hemodialysis for 1 year. A previous skin biopsy had revealed an abnormal distribution of the alpha chain of type IV collagen with the absence of the alpha 5 chain. The genetic test showed an X-linked (hemizygote) missense mutation of COL4A5 gene (c.3319 G > A; p. G1107R). The genetic analysis was extended to the other family members. His 83-year-old mother, as well as his daughter, two sisters, a niece, and two dead brothers suffered of RLS and presented the same AS mutation (Fig. 1). All the female members in the family carrying the AS mutation presented proteinuria and hematuria, with normal creatine clearance. The patient reported that his two brothers died because they were unable to undergo hemodialysis because of their severe RLS, which did not allow them to stay still long enough to carry out dialysis sessions. The patient had suffered peritoneal perforation for five times because of his need to move and inability to stay still during peritoneal dialysis; this led to the decision to shift to hemodialysis.

**Fig. 2:**
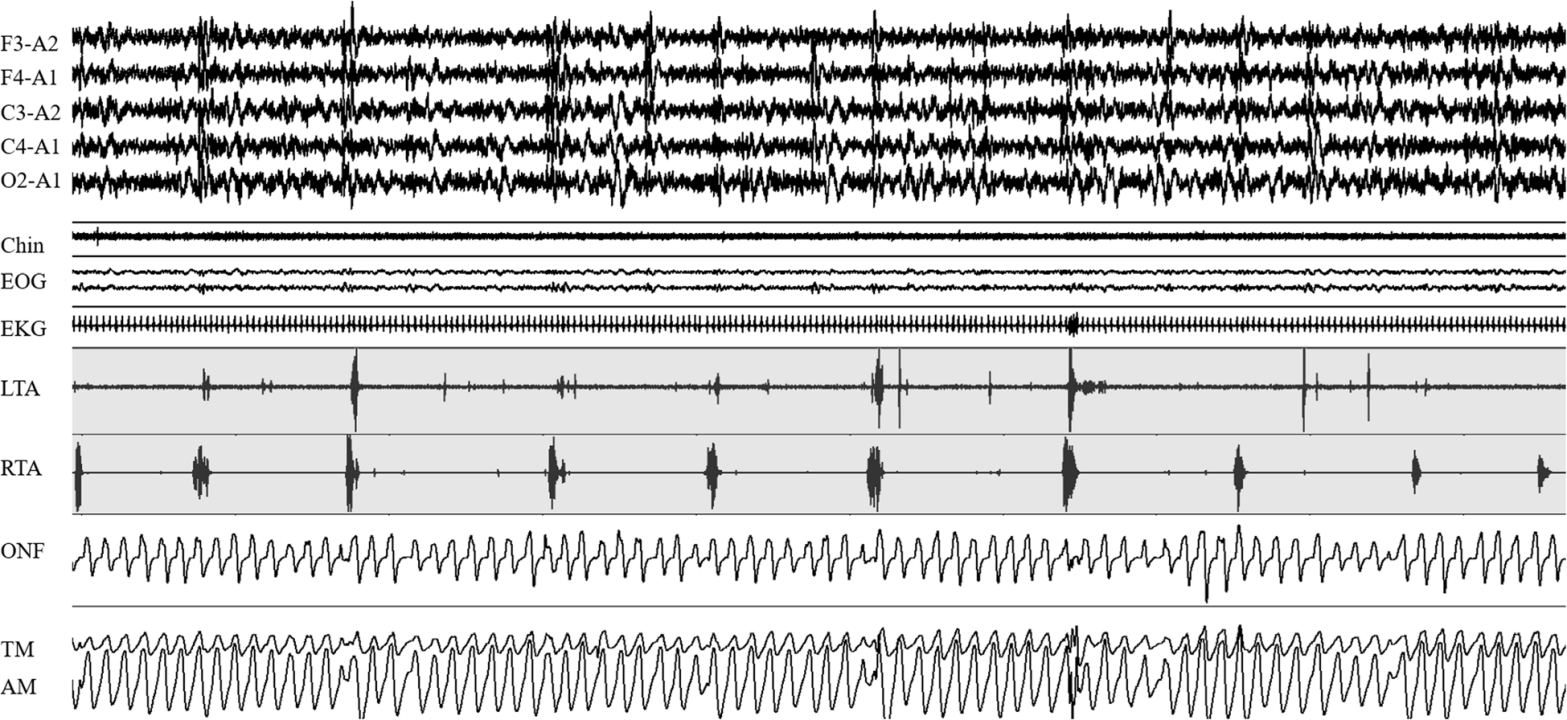
Five minutes polysomnographic sample of NREM sleep showing a sequence of periodic limb movements during sleep (grey background), recorded by surface electromyography of both left and right tibialis anterior muscles (LTA, RTA). Cortical arousals coupled with motor events visible at electroencephalic channels. F3 and F4 frontal left and right; C3 and C4 central left and right; O2 occipital right; A1 and A2 mastoid left and right; EOG = electrooculogram; Chin = electromyography of submentalis muscle; EKG = electrocardiogram; ONF = oro-nasal flow; TM = thoracic movements; AM = abdominal movements

A video-polysomnography (v-PSG) one year before our evaluation and under treatment with pramipexole 0.5 mg/day at 9 pm, showed disrupted sleep with sleep efficiency of 64 % and a PLMS index (number of PLMS per hour of sleep) of 72/hour (very severe, pathological threshold > 15/hour) (Fig. 2). Serum ferritin, transferrin saturation, vitamin D, folate and vitamin B12 were normal. During the last week preceding our evaluation, he had presented an unusual aggressive behaviour and, for the first time, he had refused his regular nephrological check-up. RLS symptoms were very severe (IRLS Rating Scale score 34, range 0–40). A clear augmentation phenomenon was diagnosed, based on the intensification of symptoms during the day, an extension of them to the upper limbs and trunk, with impossibility to rest and failed attempts to increase the dosage of pramipexole. His wife reported that the patient had several sleep attacks during the day, with one of them occurring during walking and causing a fall and a zygomatic bone fracture.

**Fig. 3:**
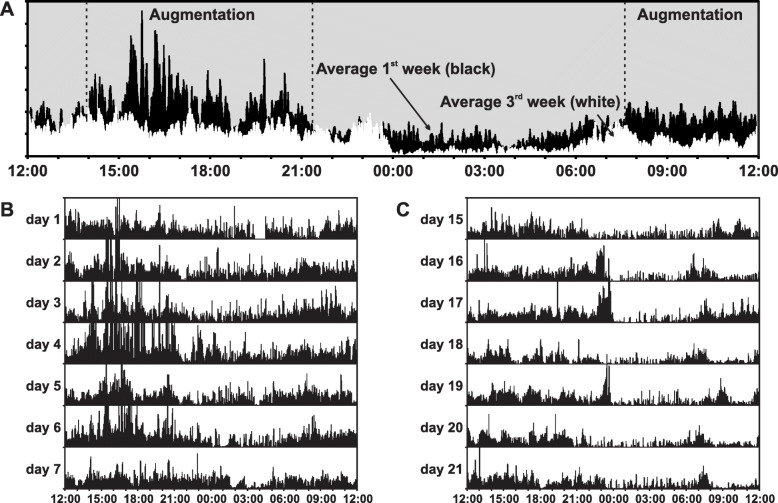
(**a**) Average actigraphic motor activity before (black area) and after (white area) drug management. (**b**) One-week actigraphic recording during treatment with immediate release pramipexole 0.75 mg/day, showing severe insomnia and high levels of motor activity during daytime. (**c**) One-week actigraphic recording after therapeutic shifting to pramipexole 0.75 mg plus clonazepam 0.5 mg, at bedtime, showing a significant improvement of both night sleep and a motor activity during daytime

A fourteen-day actigraphic recording showed a complete sleep disruption in the first week of recording (Fig. 3b). Following a modification of the treatment protocol, from pramipexole 0.75 mg/day at 9 pm to prolonged release pramipexole 0.75 mg plus clonazepam 0.5 mg, at bedtime, strongly improved his sleep-wake pattern. The improvement was documented by actigraphy in the second week of recording (Fig. 3c). The IRLS Rating Scale score decreased to 22.

One year later, the patient underwent kidney transplantation. During the procedure, the RLS therapy was acutely withdrawn, with the appearance of a severe state of motor agitation that almost compromised the surgical outcome and needed opioids in addition to the previous therapy to be controlled. Two months after transplantation, RLS symptoms considerably improved (IRLS Rating Scale score 4), and then remained stable.

A v-PSG performed 5 years after transplantation showed a normal sleep efficiency and the disappearance of PLMS. The last clinical follow-up, performed 8 years after transplantation showed a good control of symptoms obtained with pramipexole extended release 0.375 mg/day at 4 pm, rotigotine transdermal patch 3 mg/day and clonazepam 0.5 mg/day.

## Discussion and conclusions

To the best of our knowledge, this is the first observation of RLS in AS. Sleep disorders in general do not seem to be mentioned in the literature regarding the clinical picture of AS. In the present case, RLS was particularly severe, leading to a profound sleep disruption, with a major impact on the patient’s quality of life and potentially affecting the outcome of transplantation and dialysis. The development of augmentation represented an additional aggravating factor in management of RLS symptoms.

It is known that, in the long term, RLS patients treated with dopamine agonists may develop a severe drug-related complication named “augmentation”, characterized by a paradoxical response to an increase of drug dosage, a worsening and spreading of symptoms to body parts other than legs and to other periods of the day and not only at evening/night. After an average of 3 years, approximately 20 % of RLS patients develop augmentation during treatment with dopamine agonists. Augmentation represents a real challenge for sleep specialists, who often have to fall back to opioids to treat their patients [16].

The shift from an immediate to an extended-release formulation of pramipexole drastically ameliorated RLS, as already reported in augmented idiopathic RLS [17], while transplantation further improved and stabilized symptoms. Alternative treatments to avoid augmentation in ESRD patients are represented by alpha-2-delta ligands compounds, like gabapentin or pregabalin [18], as well as by non-pharmacological interventions including repetitive transcranial magnetic stimulation, compression devices, infrared therapy [19]. Of note, in a randomized placebo controlled trial, a 6-month exercise training regime was as effective as a low dosage dopamine agonist treatment in reducing RLS symptoms in hemodialysis patients [20]. Aerobic exercise training additionally improves quality of life, depression and physical performance in uremic patients [21, 22].

This case clearly shows that RLS can significantly impact and complicate the treatment of patients with AS, also increasing by far the nursing workload during hemodialysis sessions. There are at least three speculative considerations on the possible causal relationship between these two conditions that deserve to be discussed.

First, the occurrence of RLS in five females, heterozygous carriers of the AS genetic trait but with preserved renal function, seems to exclude the possibility that RLS is dependent only on renal failure in this family. However, we should take into consideration that although apparently healthy, the women with the AS mutation in our family might have been affected by a minimal kidney involvement, expressed by proteinuria and hematuria; in fact, it has been reported that females carriers of a missense mutation of the AS gene (as in our family) have a higher risk to have proteinuria than those with non-missense mutations [23]. In any case, CKD might have exacerbated RLS, explaining its higher severity in the male family members. Second, all members affected by AS or carrying the mutation were also affected by RLS. Therefore, we cannot exclude that the AS genetic mutation might be responsible also for RLS through an unknown mechanism. Despite being theoretically possible, this hypothesis does not seem to be supported by genetic studies conducted so far in RLS. The AS mutation is not included among the allelic variants significantly associated with RLS by genome wide analyses, nor among those found by linkage studies in familial RLS [24]. Moreover, in idiopathic familial RLS, symptoms are typically inherited with autosomal dominant modality and are prevalent in women. A third possibility is that the association between RLS and AS was simply incidental and a feature of this family only. Further families and patients with AS need to be screened for RLS in order to attempt to address the latter two hypotheses.

Finally, we recommend nephrologists to always carefully seek for RLS symptoms in patients with AS, because RLS is a condition that deeply affects quality of life, sleep and compliance to the dialysis. These patients need a multidisciplinary approach for an appropriate therapeutic intervention, and transplantation should be accelerated in the case of comorbidity with RLS.

## Data Availability

Data and material that support the findings of this study are available from the corresponding author, DS, upon reasonable request.

## Abbreviations

AS: Alport Syndrome.
CKD: Chronic kidney disease.
PLMS: Periodic limb movements during sleep.
RLS: Restless legs syndrome.
V-PSG: Video-polysomnography.
