# Correction to: Severe restless legs syndrome in a family with Alport syndrome

**DOI:** 10.1186/s12882-021-02501-z

**Published:** 2021-08-23

**Authors:** Davide Sparasci, Andrea Rossinelli, Raffaele Ferri, Pietro Cippà, Andrea Rinaldi, Mauro Manconi

**Affiliations:** 1 Sleep Medicine Unit, Neurocenter of Southern Switzerland, Ospedale Civico, Lugano, Switzerland; 2 Sleep Research Centre, Oasi Research Institute – IRCCS, Troina, Italy; 3 Division of Nephrology, Ente Ospedaliero Cantonale, Lugano, Switzerland; 4 Institute of Oncology Research, Faculty of Biomedical Sciences, Università della Svizzera italiana, 6500 Bellinzona, TI Switzerland; 5 Faculty of Biomedical Sciences, Università della Svizzera Italiana, Lugano, Switzerland; 6 Department of Neurology, University Hospital, Inselspital, Bern, Switzerland

**Correction to: BMC Nephrology 22, 249 (2021)**

**https://doi.org/10.1186/s12882-021-02455-2**

Following publication of the original article [1], the authors informed us that would like to add the following sentence to the Acknowledgements and Funding sections.
